# Levothyroxine dose prediction post-thyroidectomy for differentiated thyroid carcinoma

**DOI:** 10.3389/fendo.2025.1727681

**Published:** 2025-12-17

**Authors:** Li Ma, Yan Xie, Sujie Ke, Deying Liu, Linxi Wang, Lijing Wang, Libin Liu

**Affiliations:** Department of Endocrinology, Fujian Institute of Endocrinology, Fujian Medical University Union Hospital, Fuzhou, Fujian, China

**Keywords:** differentiated thyroid carcinoma, levothyroxine dose prediction, overweight and obesity, total thyroidectomy, TSH suppression therapy

## Abstract

**Objective:**

To address the overestimation of levothyroxine (L-T4) doses in conventional weight-based regimens for individuals who are overweight and obese, this study aimed to identify the most predictive body weight metrics and establish an optimized dosing model for accurate thyroid-stimulating hormone (TSH) suppression following total thyroidectomy in differentiated thyroid carcinoma (DTC).

**Methods:**

This retrospective study included 385 patients with DTC treated at our institution between October 2019 and December 2024. Patients were stratified by TSH targets (A1: <0.1 mIU/L; A2: 0.1–0.5 mIU/L; A3: 0.5–2.0 mIU/L) and body mass index (BMI) according to Chinese criteria (normal: <24 kg/m²; overweight: 24–27.9 kg/m²; obesity: ≥28 kg/m²). Linear regression analysis was used to analyze correlations between the final stable L-T4 dose and weight metrics, including total body weight, adjusted body weight, lean body weight, ideal body weight, and body surface area, followed by model validation. Model performance was internally validated using a hold-out method. Efficacy was estimated as the accuracy of the model-predicted dose compared with the actual dose required when a patient first achieved their TSH target within the first postoperative year.

**Results:**

The baseline characteristics showed no significant intergroup differences (P>0.05). Postoperative TSH levels varied significantly according to BMI (P<0.05). Patients with higher BMI required higher total L-T4 doses (µg/d) (P<0.001) but lower weight-adjusted doses (µg/kg/d) (P<0.001). Adjusted body weight best predicted L-T4 dose for patients with BMI ≤ 23.9 kg/m^2^, while lean body weight was optimal for those with BMI≥24.0 kg/m^2^. The new model achieved a significantly higher rate of accurate initial dose prediction compared with that via empirical dosing (68.0% vs. 30.2%, P<0.001).

**Conclusion:**

The BMI-stratified L-T4 dosing formula based on optimized body weight metrics demonstrated improved accuracy, expediting TSH suppression and reducing adverse events.

## 1 Introduction

Thyroid cancer has emerged as one of the fastest-growing malignancies worldwide (1, 2), with differentiated thyroid carcinoma (DTC) accounting for over 90% of cases (3, 4). Although DTC generally exhibits a favorable prognosis (5), postoperative management presents significant difficulties. Thyroid-stimulating hormone (TSH) suppression therapy is a cornerstone of DTC treatment; however, the optimization of levothyroxine (L-T4) dosing remains a clinical challenge (6, 7).

Current empirical dosing regimens (1.5–2.5 μg/kg/d or 100–150 μg/d) demonstrate substantial limitations (8–10). Ojomo et al. found that during the first postoperative follow-up, only 32% of patients were on an optimal levothyroxine (L-T4) dosage, while 26.2% were undertreated, and 41.8% were over-treated. The mean time to achieve the optimal dosage was 7.5 months, with 87.8% of patients who did not achieve optimal dosage requiring up to 1 year for dose optimization (11). Inadequate dosing may elevate the risk of recurrence, whereas excessive dosing has been associated with adverse effects, including atrial fibrillation and osteoporosis (12–14).

Obesity is a well-established independent risk factor for thyroid cancer (15), and its rising global prevalence underscores the significance of formulating L-T4 dosing regimens. From a biochemical perspective, obesity may influence L-T4 metabolism through multiple mechanisms. First, excessive adipose tissue accumulation may alter the distribution of L-T4 volume in the body. As a lipophilic hormone, L-T4 exhibits increased distribution into body fat, potentially leading to reduced plasma concentrations (16). Second, obesity-related chronic inflammation may modulate thyroid hormone metabolism via cytokines such as TNF-α and IL-6, which affect deiodinase activity and promote the conversion of T4 to reverse T3 rather than biologically active T3 (17). Furthermore, insulin resistance, a common comorbidity of obesity, may disrupt the feedback regulation of the hypothalamic pituitary–thyroid axis, further complicating thyroid hormone homeostasis (18).

Singh et al. demonstrated that patients who are overweight and obese require higher daily doses of L-T4; however, these doses are significantly lower than those calculated based on actual body weight using conventional approaches (19). This indicates that the L-T4 dosing requirements in patients who are overweight and obese are distinct and that traditional weight-based calculations may fail to achieve the target TSH levels.

To improve the accuracy of L-T4 dose prediction, multiple studies have explored this issue from various perspectives. L-T4 requirements are considered to correlate with factors such as sex, age, actual body weight, height, body mass index (BMI), estrogen therapy, calcium/iron supplementation, and gastric pH, and predictive models incorporating these variables have been proposed (20–25). Brun et al. developed a computer-assisted decision-making tool to simulate L-T4 dosing by monitoring early postoperative TSH and free thyroxine levels (10). Additionally, machine learning algorithms, such as support vector regression, have been applied to predict L-T4 doses (26). However, the existing prediction models frequently depend on complex variable combinations or require repeated testing, which limits their clinical applicability.

Most existing prediction schemes overlook the unique L-T4 dosing requirements for individuals who are overweight and obese. Studies by Papoian et al. revealed that the dose prediction accuracy is lower in this population than in individuals with normal weight (27). Santini et al. proposed that lean body weight (LBW) is the primary determinant of L-T4 requirements, particularly in patients who are overweight/obese, and that LBW-based dosing may shorten the time to achieve optimal L-T4 titration (28). Sukumar et al. further demonstrated that body surface area (BSA) was strongly correlated with L-T4 requirements than with actual weight in patients undergoing total thyroidectomy for benign thyroid disease (29). These findings suggest that weight-related metrics (e.g., adjusted weight, ideal body weight [IBW], LBW, and BSA) may better predict postoperative L-T4 doses in patients with DTC and overweight/obesity because of the influence of the latter on drug metabolism (30).

Based on this scientific rationale, we hypothesize that a BMI-stratified approach incorporating appropriate body composition metrics (e.g., LBW or BSA) can more accurately predict L-T4 requirements in patients with DTC after total thyroidectomy compared with reliance on actual body weight alone. This study, therefore, aimed to systematically evaluate the predictive value of various body weight-related indices for postoperative L-T4 dosing in DTC patients who are overweight and obese, with the goal of establishing a simplified BMI-stratified dosing model to support precise and practical TSH-suppression therapy.

## 2 Materials and methods

### 2.1 Study population

#### 2.1.1 Inclusion and exclusion criteria

Herein, 385 patients who underwent thyroidectomy at the Fujian Medical University Union Hospital between October 2019 and December 2024 were enrolled. All patients were pathologically diagnosed with DTC and received postoperative TSH-suppressive therapy. This study was conducted using a retrospective design, with the study cohort randomly split into a model development cohort (n=288) to derive the weight-based dosing formulas and an internal validation cohort (n=97) to assess the predictive accuracy of the developed models.

The inclusion criteria were as follows: (1) total thyroidectomy with pathological confirmation of DTC; (2) age >18 years; (3) postoperative TSH-suppression therapy; (4) achievement of target TSH suppression within 1 year after surgery; (5) availability of complete medical records.

The exclusion criteria were as follows: (1) a history of other malignancies; (2) dysfunction of major organs, such as the heart, liver, or kidneys; (3) pregnancy; (4) comorbidities affecting drug absorption, such as celiac disease, short bowel syndrome, ulcerative colitis, gastroparesis, and prior bariatric surgery; (5) use of medications that interfere with L-T4 absorption or metabolism, such as proton pump inhibitors, H2 receptor antagonists, bile acid sequestrants, statins, ezetimibe, ciprofloxacin, rifampin, estrogen, phenytoin, carbamazepine, or amiodarone.

This study was approved by the Medical Ethics Committee of the Fujian Medical University Union Hospital (Ethics Approval: 2023KJT001) and registered with the Chinese Clinical Trial Registry (ChiCTR Approval: 2500103665/MR-35-25-037587).

#### 2.1.2 Grouping

Based on the Guidelines for the Diagnosis and Treatment of Thyroid Nodules and Differentiated Thyroid Carcinoma (2nd Edition) (31), the 288 patients who underwent surgery for DTC were stratified into three groups according to TSH suppression targets within 1 year after surgery (1): A1 group: TSH target <0.1 mIU/L (2), A2 group: TSH target 0.1–0.5 mIU/L (3), A3 group: TSH target 0.5–2.0 mIU/L.

To assess the influence of body weight on L-T4 dosage, each group was further subdivided by BMI. BMI was calculated as weight (kg)/height² (m²) and used to classify patients into underweight/normal weight (BMI ≤23.9 kg/m²), overweight (BMI 24.0–27.9 kg/m²), and obese (BMI ≥28.0 kg/m²) groups, based on the Chinese BMI classification criteria (21).

### 2.2 Sample size estimation

Our analysis follows the widely accepted heuristic of 10 observations per predictor variable (10EPV) in linear regression, which is supported by methodological literature (32, 33). Accordingly, in conducting linear regression analyses, the sample size for each BMI subgroup was ensured to be at least 10 cases.

For the sample size estimation in the model validation phase, we planned to recruit twice as many participants in the control group as in the experimental group. Based on previously reported TSH target attainment rates of 32% (11) in the control group and 64.8% (22) in the experimental group, we conservatively set the expected rates at 32% for the control group and 50% for the experimental group for calculation purposes. Using a one-sided test with α = 0.05 and β = 0.9 (power = 90%), the estimated minimum sample sizes were 91 for the experimental group and 181 for the control group.

### 2.3 Data collection

Herein, the following demographic and clinical data were collected.

1. Baseline characteristics: sex, age, height, weight, surgical pathology, and surgical approach.
2. Laboratory parameters: free triiodothyronine, free thyroxine, TSH, total cholesterol, triglyceride, aspartate aminotransferase, alanine aminotransferase, serum creatinine, and uric acid levels.
3. L-T4 dosing and follow-up: initial daily L-T4 dose, serum free triiodothyronine, free thyroxine, TSH levels at the first follow-up, thyroid function test results, and L-T4 dose at the time of first achieving TSH suppression.

Data was anonymized to protect the privacy of patients.

### 2.4 Weight-related indices and calculations

The following weight-related parameters were calculated (34, 35).

(1) Total body weight (TBW) was assessed as the preoperative weight measured to the nearest 0.1 kg using a calibrated scale.

(2) IBW, defined as the optimal weight for health, was calculated as follows:


$$Male\;IBW\;\left(kg\right)=50+2.3\times \left(\frac{height\;\left[cm\right]}{2.54-60}\right)$$

$$Female\;IBW\;\left(kg\right)=48.67+1.65\times \left(\frac{height\;\left[cm\right]}{2.54-60}\right)$$

(3) Adjusted body weight (ABW), defined as body weight adjusted for lean mass and drug distribution volume, was calculated as follows:


ABW (kg)=IBW+0.4×(TBW−IBW)

(4) LBW, defined as the fat-free body mass, was calculated as follows:


$$Male\;LBW\;\left(kg\right)=\frac{\left(9270\times TBW\right)}{6680+216\times BMI}$$

$$Female\;LBW\;\left(kg\right)=\frac{\left(9270\times TBW\right)}{87800+244\times BMI}$$

(5) BSA was calculated using the Du Bois formula as follows:


$$BSA\;\left(m^{2}\right)=0.0061\times height\;\left(cm\right)+0.0128\times weight\;\left(kg\right)-0.1529$$

### 2.5 Statistical analysis

Statistical analyses were performed using SPSS 26.0. Regarding continuous variables, normally distributed data are expressed as mean ± standard deviation (SD) and compared using analysis of variance, while non-normally distributed data are expressed as median (P25, P75) and analyzed using the Kruskal–Wallis H test. Categorical variables are expressed as n (%) and compared using the chi-squared, continuity-adjusted chi-squared, or Fisher’s exact test, as appropriate.

Patients were stratified into BMI groups prior to model development. Within each BMI stratum, separate simple linear regression models were fitted to identify the optimal weight metric for predicting the final stable L-T4 dose (µg/day). Each weight-related index—TBW, ABW, LBW, IBW, and BSA—was evaluated as a single predictor in its own model. Model selection was based on the highest R² value among these univariable models to identify the most predictive yet parsimonious weight metric for each BMI category. Additionally, an L-T4 dose calculator was developed based on the derived model.

L-T4 dose prediction accuracy was defined as the percentage of patients whose predicted L-T4 dose was within ±12.5 µg of the actual dose required to achieve target TSH suppression. This threshold was selected based on clinical consensus, accounting for tablet formulation (minimum dose increment: 12.5 µg) and guideline-recommended adjustment ranges (36, 37). The predictive accuracy of the new model was compared with that of conventional dosing strategies using the chi-squared test. Two-tailed P-values <0.05 were considered statistically significant.

## 3 Results

### 3.1 Demographic characteristics of the study population

In this study, 288 patients who met the inclusion criteria were enrolled: 118 in A1 group (TSH target <0.1 mIU/L), 95 in the A2 group (TSH target 0.1–0.5 mIU/L), and 75 in the A3 group (TSH target 0.5–2.0 mIU/L). **Table 1** summarizes the clinical characteristics of the participants in each group. There were no significant differences in individual sociodemographic and clinical characteristics between the three TSH target groups.

**Table 1:** Comparison of Baseline Data Among Groups A1, A2, and A3.

Characteristics	A1 (n=118)	A2 (n=95)	A3 (n=75)	*P*
Gender, Female, n (%)	96 (81.4)	76 (80.0)	54 (72.0)	0.28
Age (years), Mean (SD)	47.2 ± 11.0	44.4 ± 11.6	44.9 ± 10.3	0.13
Weight (kg), Median (IQR)	60.0 (54.4, 70.0)	65.0 (58.0, 70.0)	62.5 (57.0, 74.0)	0.10
BMI (kg/m^2^), Median (IQR)	23.8 (21.4, 25.7)	24.2 (22.2, 26.8)	24.3 (21.9, 26.6)	0.25
FT_3_ (pmol/L), Median (IQR)	5.19 (4.80, 5.54)	5.24 (4.78, 5.67)	5.47 (4.76, 5.77)	0.27
FT_4_ (pmol/L), Median (IQR)	11.03 (9.97, 12.63)	11.49 (10.36, 12.31)	11.42 (10.35, 12.14)	0.56
TSH (mIU/L), Median (IQR)	2.13 (1.37, 2.79)	1.75 (1.20, 2.47)	1.65 (1.11, 2.70)	0.10
TC (mmol/L), Median (IQR)	4.86 (4.13, 5.48)	4.70 (4.11, 5.40)	4.77 (4.29, 5.58)	0.20
TG (mmol/L), Median (IQR)	1.28 (0.86, 1.86)	1.16 (0.87, 1.69)	1.25 (0.89, 2.10)	0.20
ALT (U/L), Median (IQR)	18.0 (13.0, 25.0)	16.0 (12.0, 25.0)	16.0 (10.0, 22.0)	0.75
AST (U/L), Median (IQR)	19.00 (16.0, 24.0)	18.0 (15.0, 21.0)	18.0 (15.0, 22.0)	0.38
SCr (umol/L), Median (IQR)	61.0 (54.0, 67.0)	61.0 (54.0, 68.0)	60.0 (52.0, 71.0)	0.21
UA (umol/L), Median (IQR)	311.0 (255.3, 358.3)	322.0 (284.0, 386.0)	318.0 (257.0, 380.0)	0.97

A1 group, TSH target <0.1 mIU/L; A2 group, TSH target 0.1–0.5 mIU/L; A3 group, TSH target 0.5–2.0 mIU/L.

BMI, body mass index; FT3, free triiodothyronine; FT4, free thyroxine; TSH, thyroid-stimulating hormone (ultrasensitive assay); TC, total cholesterol; TG, triglycerides; AST, aspartate aminotransferase; ALT, alanine aminotransferase; SCr, serum creatinine; UA, uric acid.

### 3.2 Postoperative TSH control levels in different BMI categories

Significant differences were observed in the TSH control levels at the first postoperative follow-up among patients with DTC stratified by BMI (P = 0.025).

In the BMI ≤23.9 kg/m² group (n=140), 32.9% (46/140) of patients achieved target TSH levels, while 40.7% (57/140) had TSH levels below the target range. The BMI 24.0–27.9 kg/m² group (n=108) had a lower TSH target achievement rate of 25.0% (27/108), with 75.0% (81/108) demonstrating TSH levels below or above the desired range. Although the BMI ≥28.0 kg/m² group (n=40) had a relatively higher target achievement rate of 35.0% (14/40), 65.0% (19/40) failed to maintain TSH levels within the recommended range. The detailed data is presented in **Table 2**.

**Table 2:** Comparison of Thyroid Function Status at Initial Dose Across Different BMI Categories.

BMI	TSH below target range (n/%)	TSH within target range (n/%)	TSH above target range (n/%)	*P*
BMI Categories				0.03
≤23.9 (kg/m^2^)	57 (40.7%)	46 (32.9%)	37 (26.4%)	
24.0-27.9 (kg/m^2^)	41 (38.0%)	27 (25.0%)	40 (37.0%)	
≥28.0 (kg/m^2^)	7 (17.5%)	14 (35.0%)	19 (47.5%)	

Underweight/normal, BMI ≤23.9 kg/m²; Overweight, BMI 24.0-27.9 kg/m²; Obese, BMI ≥28.0 kg/m².

TSH below target range, TSH level lower than the TSH suppression target range.

TSH within target range, TSH level within the TSH suppression target range.

TSH above target range, TSH level higher than the TSH suppression target range.

### 3.3 Comparison of daily L-T4 dosage across BMI categories in patients with controlled TSH levels

Among patients who achieved the target TSH levels, significant variations in L-T4 dosing were observed between the different BMI categories (P<0.001). Patients who were obese (BMI ≥28.0 kg/m²) required significantly higher absolute daily L-T4 doses than did those who were overweight (BMI 24.0–27.9 kg/m²) and underweight/normal weight (BMI ≤23.9 kg/m²), with the differences being statistically significant (P<0.001).

However, while the absolute L-T4 requirement increased with BMI, the daily stable weight-adjusted dose (daily L-T4 dosage/weight, μg/kg/day) showed a progressive decrease across BMI categories (P<0.001). This inverse relationship between BMI and the daily stable weight-adjusted dose was statistically significant, suggesting that dose calculations based solely on body weight may require adjustments for patients with a higher BMI. The detailed data is presented in **Table 3**.

**Table 3:** Comparison of Daily Stable Dose and Daily Stable Weight- Adjusted Dose of L-T_4._.

Groups	dose	Underweight/normal	Overweight	Obese	*P*
A1	daily L-T4 doses (µg/d)	100.0 (100.0, 107.1)	110.7 (107.1, 117.9)	125.0 (121.4, 135.7)	<0.001
	daily stable weight-adjusted dose (µg/kg/d)	1.9 (1.8, 2.0)	1.7 (1.6, 1.8)	1.6 (1.5, 1.6)	<0.001
A2	daily L-T4 doses (µg/d)	100.0 (92.9, 104.5)	103.6 (100.0, 117.9)	114.3 (101.8, 127.7)	<0.001
	daily stable weight-adjusted dose (µg/kg/d)	1.8 (1.7, 1.9)	1.6 (1.5, 1.7)	1.5 (1.4, 1.6)	<0.001
A3	daily L-T4 doses (µg/d)	100.0 (100.0, 106.3)	114.3 (100.0, 128.6)	121.4 (114.3, 125.0)	<0.001
	daily stable weight-adjusted dose (µg/kg/d)	1.8 (1.7, 1.9)	1.7 (1.6, 1.7)	1.5 (1.4, 1.6)	<0.001

A1 group, TSH target <0.1 mIU/L; A2 group, TSH target 0.1–0.5 mIU/L; A3 group, TSH target 0.5–2.0 mIU/L.

Underweight/normal, BMI ≤23.9 kg/m²; Overweight, BMI 24.0-27.9 kg/m²; Obese, BMI ≥28.0 kg/m².

### 3.4 Linear regression analysis

Linear regression models were developed to predict the L-T4 dosage based on various weight-related parameters, including TBW, ABW, IBW, LBW, and BSA. The optimal predictive formulae were selected based on their coefficients of determination (R²) and are presented below. In the equations, “y” represents the daily L-T4 dose in μg.

#### 3.4.1 TSH suppression target <0.1 mIU/L

For patients with BMI ≤23.9 kg/m², the equation was as follows: y=7+1.8× ABW (R² = 0.722).

For patients with BMI 24.0-27.9 kg/m², the equation was as follows: y=75+0.85× LBW (R² = 0.850).

For patients with BMI ≥28.0 kg/m², the equation was as follows: y=95+0.63× LBW (R² = 0.832).

Based on these findings, the ABW-based formula is recommended for patients with BMI ≤23.9 kg/m², while the LBW-based formula is preferred for those with BMI ≥24.0 kg/m² (**Table 4**).

**Table 4:** Linear regression analysis.

weight-related parameters	A1			A2			A3		
	Underweight/ normal (n=61)	Overweight (n=43)	Obese (n=14)	Underweight/ normal (n=42)	Overweight (n=37)	Obese (n=16)	Underweight/ normal (n=36)	Overweight (n=29)	Obese (n=10)
TBW	26 + 1.45×kg	51 + 0.92×kg	85 + 0.52×kg	26 + 1.31×kg	30 + 1.17×kg	-5 + 1.56×kg	13 + 1.57×kg	13 + 1.46×kg	83 + 0.45×kg
	(0.164)	(0.090)	(0.180)	(0.174)	(0.186)	(0.343)	(0.267)	(0.218)	(0.230)
(R-sq)	0.571	0.715	0.410	0.584	0.531	0.598	0.503	0.625	0.326
ABW	7 + 1.8×kg	54 + 0.97×kg	81 + 0.68×kg	1 + 1.75×kg	31 + 1.28×kg	-15 + 2.02×kg	-15 + 2.10×kg	19 + 1.53×kg	71 + 0.71×kg
	(0.146)	(0.070)	(0.133)	(0.143)	(0.161)	(0.308)	(0.261)	(0.214)	(0.173)
(R-sq)	0.722	0.824	0.685	0.790	0.643	0.756	0.655	0.654	0.676
IBW	10 + 1.75×kg	60 + 0.94×kg	87 + 0.69×kg	4 + 1.72×kg	39 + 1.25×kg	6 + 1.96×kg	-1 + 1.86×kg	26 + 1.52×kg	73 + 0.77×kg
	(0.146)	(0.064)	(0.101)	(0.145)	(0.149)	(0.327)	(0.277)	(0.214)	(0.111)
(R-sq)	0.710	0.841	0.795	0.778	0.665	0.719	0.571	0.650	0.857
LBW	46 + 1.60×kg	75 + 0.85×kg	95 + 0.63×kg	40 + 1.56×kg	58 + 1.15×kg	42 + 1.55×kg	36 + 1.74×kg	49 + 1.41×kg	87 + 0.63×kg
	(0.131)	(0.055)	(0.081)	(0.129)	(0.127)	(0.217)	(0.252)	(0.172)	(0.089)
(R-sq)	0.715	0.850	0.832	0.785	0.702	0.785	0.582	0.713	0.862
BSA	27 + 86.85×m²	30 + 48.47×m²	68 + 30.97×m²	-25 + 79.95×m²	63.99×m²	-54 + 93.25×m²	-54 + 99.94×m²	-20 + 78.18×m²	62 + 30.58×m²
	(8.168)	(4.366)	(8.766)	(8.708)	(9.547)	(18.468)	(13.576)	(11.837)	(11.832)
(R-sq)	0.657	0.750	0.510	0.678	0.562	0.646	0.614	0.618	0.455

A1 group, TSH target <0.1 mIU/L; A2 group, TSH target 0.1–0.5 mIU/L; A3 group, TSH target 0.5–2.0 mIU/L.

Underweight/normal, BMI ≤23.9 kg/m²; Overweight, BMI 24.0-27.9 kg/m²; Obese, BMI ≥28.0 kg/m².

Standard errors of the coefficients are given in parentheses, TBW, total body weight; ABW, adjusted body weight; IBW, ideal body weight; LBW, lean body weight; BSA, body surface area.

#### 3.4.2 TSH suppression target 0.1-0.5 mIU/L

For patients with BMI ≤23.9 kg/m², the equation was as follows: y=1+1.75×ABW (R² = 0.790).

For patients with BMI 24.0-27.9 kg/m², the equation was as follows: y=58+1.15×LBW (R² = 0.702).

For patients with BMI ≥28.0 kg/m², the equation was as follows: y=42+1.55×LBW (R² = 0.785).

Similar to the previous TSH suppression target range, for a TSH suppression target of 0.1–0.5 mIU/L, the ABW-based formula is recommended for patients with a BMI ≤23.9 kg/m², while the LBW-based formula is preferred for those with a BMI ≥24.0 kg/m² (**Table 4**).

#### 3.4.3 TSH suppression target 0.5-2.0 mIU/L

For patients with BMI ≤23.9 kg/m², the equation was as follows: y=−15+2.10×ABW (R² = 0.655).

For patients with BMI 24.0-27.9 kg/m², the equation was as follows: y=49+1.41×LBW (R² = 0.713).

For patients with BMI ≥28.0 kg/m², the equation was as follows: y=87+0.63×LBW (R² = 0.862).

Consistent with other ranges, for a TSH suppression target of 0.5–2.0 mIU/L, ABW-based calculations are recommended for normal-weight patients, and LBW-based calculations are recommended for overweight/obese patients (**Table 4**).

### 3.5 Model validation

#### 3.5.1 Baseline characteristics

Comparative analysis revealed no statistically significant differences (P>0.05) in sex distribution, age, or TSH target stratification between the empirical scheme group (n=288) and the new scheme group (n=97), as detailed in **Supplementary Table S1**.

#### 3.5.2 Dosing efficacy outcomes

**Figure 1:**
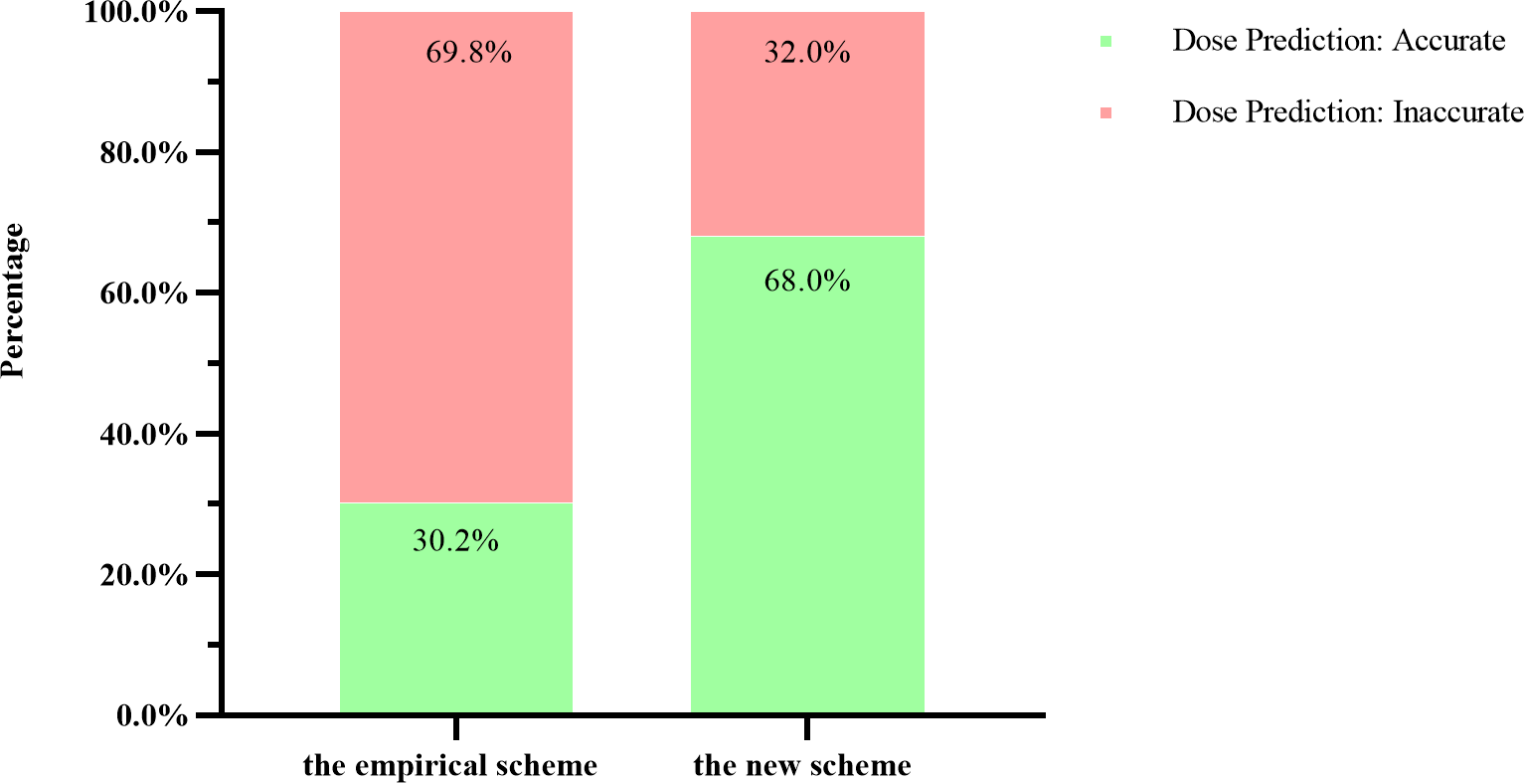
Comparison of initial dose accuracy between the empirical scheme and the new scheme group.

In the empirical scheme group, the initial L-T4 dose was accurate (i.e., within the predefined acceptable range of the final stable dose) in 87 patients (30.2%), whereas in the new scheme group, the initial dose was accurate in 66 patients (68.0%). This corresponds to a 37.8% absolute reduction in the rate of inaccurate initial dosing (32.0% in the new scheme group vs. 69.8% in the empirical scheme group). A χ² test (all expected frequencies >5; minimum expected frequency=87) revealed a statistically significant difference between the groups (χ² = 43.370, *P* < 0.001), indicating that the new predictive model significantly improved the accuracy of the initial L-T4 dose estimation (**Figure 1**).

### 3.6 Web-based calculator

We developed an online tool to implement the new dosing algorithm, in which users can input height, weight, and TSH targets to obtain personalized L-T4 dose estimates. The tool incorporates automated dose calculation using validated BMI-stratified formulas and a built-in TSH target assessment scale based on clinical guidelines (28).

Currently, the tool is accessible only in the internal network at http://[internal-IP]/lt4/LT4calculationtool.html

This tool standardizes dose determination and improves clinical implementation of our model.

## 4 Discussion

Our study revealed important BMI-dependent variations in L-T4 treatment response. Patients with higher BMI showed a greater proportion of TSH levels above the target range, whereas those with a lower BMI had TSH levels below the target range more frequently. This pattern suggests a relative underdosing in patients were overweight and obese in our initial L-T4 regimen. Interestingly, these findings appear contradictory to those of previous studies that reported higher rates of thyrotoxicosis in patients with elevated BMI (19). This discrepancy likely stems from fundamental differences in dosing protocols between studies; while previous studies used weight-adjusted calculations (1.5-2.5 μg/kg) (22), our institution primarily employed fixed doses (mostly 100 μg/day), potentially explaining the observed underdosing in patients who were overweight/obese.

To better understand these observations, we systematically analyzed the relationship between body weight and L-T4 dosage requirements. Our analysis revealed that while the absolute daily L-T4 dose (μg/day) increased progressively with higher BMI, the weight-adjusted daily dose (μg/kg/day) demonstrated a gradual decrease. These findings, which are consistent with those of previous reports (19, 27, 38, 39), underscore the unique L-T4 dosing requirements of patients who are overweight and obese. This is particularly relevant given the parallel trends between the rising prevalence of thyroid cancer and obesity in China (40).

Building on prior work on benign thyroid diseases that identified LBW and BSA as superior predictors of L-T4 requirements (41, 42), we extended this investigation to patients with DTC who had undergone surgical treatment. Our comprehensive evaluation of weight-adjusted parameters revealed that in patients with normal weight (BMI ≤23.9 kg/m²), ABW showed the strongest correlation (R²=65.5%-79.0%), while in patients who were overweight/obese (BMI≥24.0 kg/m²), LBW demonstrated superior predictive value (R²=70.2%-86.2%). These results, consistent with previous findings (28, 43, 44), likely reflect fundamental differences in body composition and L-T4 distribution patterns across BMI categories.

The superiority of the LBW as a predictive parameter supports its physiological relevance. Using dual-energy X-ray absorptiometry analysis, Santini et al. (45) demonstrated that LBW, which represents metabolically active fat-free mass, best correlates with L-T4 requirements. This association likely reflects the central role of lean tissues in thyroxine metabolism, where type 2 deiodinase (muscle) and type 1 deiodinase (liver) mediate its conversion to active triiodothyronine (46, 47). From a biochemical perspective, LBW more accurately represents the volume of metabolically active tissues compared with TBW, while adipose tissue contributes minimally to L-T4 distribution and exhibits lower clearance rates (16). Clinical validation comes from studies on bariatric surgery showing that while total L-T4 requirements decrease postoperatively, the dose per kilogram of LBW remains stable, confirming the pharmacological irrelevance of fat mass (48). This tissue-specific difference explains why LBW-based models demonstrate superior predictive accuracy in individuals with obesity.

Translating these findings into clinical practice, our novel L-T4 dosing algorithm demonstrated significantly improved performance (initial TSH target attainment: 68% with the new predictive model vs. 30.2% with the conventional dosing regimen) while maintaining practicality. This result aligns with the 60–80% success rate reported for other predictive models (25). Unlike previously proposed methods that incorporate numerous variables such as age, sex, calcium/iron/vitamin supplementation, and tumor stage (9, 36, 49), our model relies only on core parameters: height, adjusted body weight, lean body weight, and TSH target range. The use of formula-based calculations for ABW and LBW—rather than instrument-based body composition analysis—enhances its feasibility for widespread clinical adoption. To address variations in regression coefficients and R² values across different TSH targets and BMI categories, we developed a stratified dosing strategy. Furthermore, to maximize operational efficiency, we implemented a web-based calculator that integrates real-time computation of ABW and LBW along with TSH target configuration, allowing clinicians to obtain personalized L-T4 dose recommendations by simply entering three basic parameters: height, body weight, and TSH target. Notably, by improving first-dose accuracy, our model significantly reduces the need for repeated dose adjustments, thereby potentially shortening the time required to achieve target TSH levels compared to that via conventional methods, which reportedly require a median duration of 14.5 weeks (interquartile range: 2–120 weeks) (50, 51). While prospective validation is required to quantify the exact time reduction, our model demonstrates potential to accelerate TSH target achievement. Current evidence supports achieving TSH suppression within 6–12 months postoperatively, as delayed suppression beyond the first year is associated with an increased risk of recurrence (6). Conversely, chronic over-suppression should be avoided given its established associations with atrial fibrillation and bone loss (13, 14).

Despite encouraging results, several limitations warrant consideration. The single-center retrospective design may have introduced selection bias, and while the sample size satisfied initial analytical requirements, further expansion in future studies is necessary. Additionally, the exclusion of patients with significant comorbidities limits our understanding of the model’s accuracy in these complex populations, potentially creating selection bias in clinical applications. Additionally, it should be noted that surgical factors such as thyroidectomy extent and postoperative parathyroid function status may influence L-T4 requirements; however, these variables were not systematically evaluated in this study. To mitigate these limitations, future studies should adopt multicenter prospective designs, expand sample sizes, and develop adjustment factors for specific subgroups (e.g., older patients and those with gastrointestinal diseases). Moreover, the underlying molecular mechanisms require further elucidation.

## 5 Conclusion

This study establishes a BMI-stratified L-T4 dosing strategy for postoperative DTC patients and demonstrates the superior efficacy of LBW-based calculations in individuals who are overweight or obese. Our web-based calculator integrates these findings while balancing precision and clinical practicality. Although multicenter validation is needed, this approach addresses critical gaps in personalized thyroid cancer management, particularly within the context of China’s obesity epidemic. Our model enables clinicians to achieve more accurate initial dosing using readily available metrics, reduces dosage adjustment frequency, accelerates TSH target achievement, and ultimately, provides safer, more effective long-term management for patients with DTC.

## Data Availability

The raw data supporting the conclusions of this article will be made available by the authors, without undue reservation.
