# Supplementary material for: Levothyroxine dose prediction post-thyroidectomy for differentiated thyroid carcinoma
Source: Front Endocrinol (Lausanne). 2025 Dec 17;16:1727681. doi: 10.3389/fendo.2025.1727681 (PMC12753355; doi:10.3389/fendo.2025.1727681)
Supplement: Supplementary file 1 [file Table1.doc]

Table S1: Comparison of basic data between the empirical scheme group and the new scheme group

|  | the empirical scheme group（n=288） | the new scheme group（n=97） | *P* |
| --- | --- | --- | --- |
| Gender, Female, n（%） | 226（78.5） | 69（71.1） | 0.14 |
| Age（years）, Mean (SD) | 45.7±11.0 | 45.7±12.5 | 0.06 |
| TSH target stratification，n（%） |  |  | 0.06 |
| ＜0.1(mIU/L) | 118（41.0） | 53（54.6） |  |
| 0.1-0.5(mIU/L) | 95（33.0） | 25（25.8） |  |
| 0.5-2.0(mIU/L) | 75（26.0） | 19（19.6） |  |
